# Comparison of prolonged postoperative ileus between laparoscopic right and left colectomy under enhanced recovery after surgery: a propensity score matching analysis

**DOI:** 10.1186/s12957-022-02504-6

**Published:** 2022-03-04

**Authors:** Zhenmeng Lin, Chunkang Yang, Yi Wang, Mingfang Yan, Huizhe Zheng

**Affiliations:** 1 Department of Gastrointestinal Surgery, Fujian Medical University Cancer Hospital & Fujian Cancer Hospital, No 420 fuma road, Jin’ an district, Fuzhou, China; 2 Department of Anesthesiology Surgery, Fujian Medical University Cancer Hospital & Fujian Cancer Hospital, No 420 fuma road, Jin’ an district, Fuzhou, China

**Keywords:** Enhanced recovery after surgery, Prolonged postoperative ileus, Laparoscopic colectomy, Colon cancer

## Abstract

**Background:**

There were differences in the recovery of bowel function and prolonged postoperative ileus (PPOI) between laparoscopic right colectomy (RC) and left colectomy (LC) under the guidance of enhanced recovery after surgery.

**Methods:**

We selected 870 patients who underwent elective laparoscopic colectomy from June 2016 to December 2021, including 272 patients who had RC and 598 who had LC. According to 1:1 proportion for propensity score matching and correlation analysis, 247 patients who had RC and 247 who had LC were finally enrolled.

**Results:**

The incidence of PPOI in all patients was 13.1%. Age, sex, smoking habit, preoperative serum albumin level, operation type, and operation time were the important independent risk factors based on multivariate logistic regression and correlation analysis for PPOI (*p*<0.05). Age, sex, body mass index, preoperative serum albumin level, operation time, and degree of differentiation between the two groups were significantly different before case matching (*p*<0.05). There were no statistically significant differences in baseline characteristics and preoperative biochemical parameters between the two groups after case matching (*p*>0.05). The incidence of PPOI in patients who had RC was 21.9%, while that in patients who had LC was 13.0%. The first flatus, first semi-liquid, and length of stay in LC patients were lower than those in RC patients (*p*<0.05).

**Conclusion:**

The return of bowel function in LC was faster than that in RC, and the incidence of PPOI was relatively lower. Therefore, caution should be taken during the early feeding of patients who had laparoscopic RC.

## Introduction

Postoperative ileus (POI) is defined as an inevitable, temporary decrease in gastrointestinal motility following major abdominal surgery. The average gut dysmotility is widely reported to last 0–24 h in the small intestine, 24–48 h in the stomach, and 48–72 h in the colon [1, 2]. Prolonged postoperative ileus (PPOI) is a gastrointestinal function that does not recover beyond this time. The principal manifestation of PPOI is nausea and vomiting, abdominal distension, pain, inability to eat or drink, and delayed passage of flatus and stool, which lead to increased postoperative complications, slow rehabilitation, extended length of hospital stay, aggravating financial burden, and increased mortality risk [3, 4].

The enhanced recovery after surgery (ERAS) program aims to promote early feeding and progressive patient mobilization, shorten the length of hospital stay, and improve the quality of life of patients without affecting the overall survival and relapse-free survival [5–7]. ERAS has been rapidly popularized and applied in recent years. In 2017, the American Society of Colon and Rectal Surgeons (ASCRS) and the Society of American Gastrointestinal and Endoscopic Surgeons (SAGES) jointly released the clinical practice guideline of ERAS in colorectal surgery to provide important references and guidance for clinical work [8]. There are obvious differences in clinicopathological features, molecular biology, histological features, and prognosis between left and right colon tumors [9, 10]. Moreover, there are differences in the return of bowel function and complications after surgery [11]. However, the ERAS clinical practice guideline does not distinguish between right colectomy (RC) and left colectomy (LC). Therefore, the present study aimed to compare the incidence of PPOI and postoperative return of bowel function between laparoscopic RC and LC with the ERAS protocol.

## Methods

### Patients

Patients who underwent elective laparoscopic colectomy at Fujian Cancer Hospital from June 2016 to December 2021 were selected for the study. The inclusion criteria were as follows: (1) pathologically confirmed colon cancer; (2) radical resection; (3) adequate implementation of more than 70% compliance with the ERAS protocol (The ERAS pathway is shown in Table 1); and (4) complete clinical characteristics. The exclusion criteria were as follows: (1) emergency surgery for preoperative intestinal hemorrhage and ileus; (2) multi-organ resection combined with colorectal cancer; (3) mental disturbance; (4) temporary ileostomy; and (5) conversion to open surgery during the operation.

**Table 1:** ERAS protocol applied in the study

Preoperative period	Intraoperative period	Postoperative period
1. Preoperative assessment and education 2. Mechanical bowel preparation is not to be used routinely (according to guidelines) 3. Nutritional assessment and enteral nutrition support throughout the day before the surgery 4. Low molecular weight heparin or compression stockings for thromboprophylaxis 5. Preoperative fasting for clear liquids (water, coffee, juice without pulp) limited to 2 h and 6 h for solids 6. Drink 50 g of carbohydrate in at least 400 mL of fluid up to 2 h before the induction of anesthesia	7. Antibiotic prophylaxis given 30–60 min before the first incision 8. Intraoperative goal-directed fluid therapy 9. Intraoperative warming for the prevention of intraoperative hypothermia 10. Try to put as little intra-abdominal drains as possible	11. Removal of nasogastric tubes immediately after the surgery 12. Restrictive perioperative fluid management after the surgery 13. Remove the catheter 24 h after the surgery 14. Prevention of postoperative nausea and vomiting (5-HT3 receptor antagonist + dexamethasone +metoclopramide) 15. A multimodal, opioid-sparing, pain management plan was employed and implemented 16. Early oral feeding (drink water 2 h after the surgery) and soft-food diet on the second postoperative day 17. Early mobilization (sit in a chair at 2 h after the procedure. Out-of-bed activity on the first postoperative day).

*5-HT3* 5-hydroxytryptamine subtype 3

### Definitions

RC is defined as the removal of the terminal ileum and the ascending right colon followed by an ileo-colonic anastomosis, including cecal, ascending, and hepatic flexure colon cancers.

LC is defined as the resection of the descending colon or the sigmoid colon followed by a colo-colonic anastomosis, including splenic flexure, descending, sigmoid, and rectosigmoid junction colon cancers [12, 13]. During the study period, 870 patients with colon cancer were included as research subjects, including 598 who had LC and 272 who had RC.

The definition of PPOI has not been unified yet. According to references, PPOI is defined as meeting two or more of the following criteria, which were assessed on or after the fourth day of the postoperative period [14, 15]: 1. nausea or vomiting, 2. inability to tolerate an oral diet over the last 24 h, 3. abdominal distention, 4. absence of flatus over the last 24 h, and 5. ileus noted on plain abdominal films or computed tomography scans.

### Statistical analysis

The SPSS 24.0 software was used for all statistical analyses. The continuous data were evaluated using the Student’s test or Mann–Whitney *U* test. The chi-square test was used to compare the count data of the two groups. Univariate analysis was performed to identify risk factors associated with PPOI. Variables with *p* <0.05 in the univariate analyses were analyzed in the subsequent multivariable logistic regression model. The nearest neighbor matching method was used for 1:1 matching, and the caliper value was 0.05. Statistical significance was set at *p* <0.05.

## Results

### Postoperative outcomes of patients

PPOI occurred in 13.1% (114/870) of all patients with colon cancer. The average length of postoperative hospital stay in patients with PPOI was 8.6±2.5 days, which was significantly longer than the 6.3±1.1 days in patients without no PPOI before case matching (*t*=12.049, *p*=0.000).

### Univariate and multivariate analysis of PPOI risk factors in colon cancer

Among the same background and comorbidity measures, sex, age, smoking habit, preoperative serum albumin level, operation type, and operation time were associated with PPOI (Table 2). All these variables were entered in the multivariate logistic regression model. Multivariate analysis found the following independent risk factors for PPOI: sex (odds ratio [OR]=2.242), age (OR=0.935), smoking habit (OR=1.732), preoperative serum albumin level (OR=1.072), operation type (OR=2.193), and operation time (OR=2.205) (Table 3).

**Table 2:** Univariate analysis for PPOI risk factors in colon cancer

Variable	PPOI (*n*=114)	NO PPOI (*n*=756)	Univariate OR (95% CI)	*p* value
Sex			1.923 (1.242–2.977)	0.003
Male	83	440		
Female	31	316		
Age (years)	64.4±10.3	55.6±12.0	0.932 (0.913–0.950)	0.000
Diabetes mellitus			0.777 (0.517–1.170)	0.227
No	71	514		
Yes	43	242		
Hypertension			0.859 (0.569–1.296)	0.469
No	73	510		
Yes	41	246		
Smoking habit			1.830 (1.211–2.766)	0.004
No	71	568		
Yes	43	188		
Current alcohol use			0.774 (0.511–1.172)	0.227
No	74	533		
Yes	40	223		
Previous abdominal surgery			0.741 (0.438–1.254)	0.264
No	94	653		
Yes	20	103		
Chronic obstructive pulmonary disease			0.723 (0.412–1.269)	0.258
No	97	671		
Yes	17	85		
BMI (kg/m^2^)	22.8±2.5	23.1±3.2	1.043 (0.977–1.113)	0.207
Preoperative anemia			0.817 (0.532–1.256)	0.358
No	79	555		
Yes	35	201		
Preoperative serum albumin level (g/L)	37.7±3.7	39.0±4.6	1.070 (1.022–1.120)	0.004
ASA score			0.728 (0.476–1.114)	0.143
I–II	77	560		
III–IV	37	196		
Tumor size			0.768 (0.517–1.141)	0.191
<5 cm	51	388		
≥5 cm	63	368		
Operation type			2.414 (1.619–3.600)	0.000
LC	58	540		
RC	56	216		
Operation time (min)			1.799 (1.196–2.707)	0.005
<180	41	380		
≥180	73	376		
Intraoperative blood loss (ml)	179.8±101.6	169.3±72.7	0.998 (0.986–1.011)	0.178
Grade of differentiation			0.997 (0.739–1.364)	0.987
Well	22	119		
Moderate	56	426		
Poorly	36	211		
Lymph node resected	23.5±9.3	24.3±9.6	1.010 (0.988–1.032)	0.395
pTNM stage			1.022 (0.774–1.349)	0.880
I	23	124		
II	46	354		
III	45	278		

*PPOI* prolonged postoperative ileus, *OR* odds ratio, *BMI* body mass index, *ASA* American Society of Anesthesiologists, *LC* left colectomy, *RC* right colectomy

**Table 3:** Multivariate analysis of PPOI after colon cancer surgery

Parameter	*β*	Std. error	Wald chi-square	*p* value	Odds ratio	95% CI for odds ratio
Age	− 0.068	0.011	40.126	0.000	0.935	0.915–0.954
Sex	0.807	0.239	11.382	0.001	2.242	1.403–3.583
Smoking habit	0.549	0.228	5.820	0.016	1.732	1.109–2.706
Preoperative Serum albumin level	0.069	0.026	7.042	0.008	1.072	1.018–1.128
Operation type	0.785	0.222	12.476	0.000	2.193	1.418–3.390
Operation time	0.791	0.227	12.185	0.000	2.205	1.415–3.438

*95%CI *95% confidence interval

### Comparison of the type of surgical procedure of patients who had RC

The occurrence probability of PPOI in patients who had laparoscopic right colectomy (LRC) was higher than that in patients who had totally laparoscopic right colectomy (TLRC). Compared with LRC, TLRC was associated with shorter first flatus, first semi-liquid diet, and postoperative length of stay (*p*<0.05) (Table 4).

**Table 4:** Comparison of postoperative gastrointestinal function recovery and discharge time between LRC and TLRC

Group	LRC (*n*=181 )	TLRC(*n*= 91 )	*t*/*χ*^2^	*p* value
PPOI			7.707	0.005
No	135	81		
Yes	46	10		
First flatus (days)	3.8±2.6	2.8±1.9	3.009	0.003
First semi-liquid diet (days)	5.3±2.6	4.4±2.0	3.011	0.003
Postoperative length of stay (days)	7.8±2.8	7.1±1.7	2.239	0.026

*LRC* laparoscopic right colectomy, *TLRC* totally laparoscopic right colectomy

### Comparison of general clinicopathological data of patients who had RC and LC before matching

Compared with patients who had RC, statistical differences were observed in age, sex, body mass index BMI, preoperative serum albumin level, operation time, and grade of differentiation in patients who had LC before case matching. The incidence of PPOI in patients who had RC was 20.6% (56/272), which was higher than the 9.7% incidence (58/598) in patients who had LC. The first flatus, semi-liquid diet, and length of postoperative hospital stay in patients who had RC were higher than those in patients who had LC (Table 5).

**Table 5:** Demographic characteristics of patients and postoperative functional recovery before matching

Variable	LC (*n*=598)	RC (*n*=272)	*t*/*χ*^2^	*p* value
Sex			4.073	0.044
Male	373	150		
Female	225	122		
Age (years)	55.8±11.7	58.8±12.7	− 3.458	0.001
Diabetes mellitus			0.204	0.652
No	405	180		
Yes	193	92		
Hypertension			0.672	0.412
No	406	177		
Yes	192	95		
Smoking habit			0.212	0.645
Nonsmoker	442	197		
Smoker	156	75		
Current alcohol use			0.846	0.358
No	423	184		
Yes	175	88		
Previous abdominal surgery			0.093	0.760
No	512	235		
Yes	86	37		
Chronic obstructive pulmonary disease			0.064	0.801
No	529	239		
Yes	69	33		
BMI (kg/m^2^)	23.3±3.2	22.7±2.7	2.416	0.016
Preoperative anemia			1.826	0.177
No	444	190		
Yes	154	82		
Preoperative serum albumin level	39.1±4.5	38.1±4.4	3.055	0.002
ASA			0.725	0.395
I–II	443	194		
≥III	155	78		
Tumor size			0.387	0.534
<5 cm	306	133		
≥5 cm	292	139		
Operation time (min)			5.744	0.017
<180	273	148		
≥180	325	124		
Blood loss (ml)	168.9±76.2	174.7±79.1	− 1.039	0.299
Grade of differentiation			12.860	0.002
Well	92	49		
Moderate	355	127		
Poorly	151	96		
Lymph node resected	23.9±9.6	24.9±9.4	-1.527	0.127
pTNM stage			3.808	0.149
I	103	44		
II	262	138		
III	233	90		
PPOI			19.470	0.000
No	540	216		
Yes	58	56		
First flatus	2.1±1.8	3.5±2.4	− 9.379	0.000
First semi-liquid diet	3.6±1.8	5.0±2.5	− 9.765	0.000
Length of stay	6.2±1.4	7.6±2.3	− 9.674	0.000

*LC* left colectomy, *RC* right colectomy, *BMI* body mass index, *ASA* American Society of Anesthesiologists, *PPOI* prolonged postoperative ileus

### Clinicopathological characteristics after case matching

To reduce the possibility of selection bias, we conducted propensity score matching. Based on baseline data, the propensity score matching method was conducted with 1:1 matching, and 247 patients were included in each group. The clinicopathological features between the two groups were not statistically significantly different after matching (Table 6).

**Table 6:** Comparison of the two groups’ baseline characteristics after matching

Variable	LC (*n*=247)	RC (*n*=247 )	*t*/*χ*^2^	*p* value
Sex			0.206	0.650
Male	143	138		
Female	104	109		
Age (years)	57.5±12.2	59.1±12.9	− 1.353	0.177
Diabetes mellitus			0.037	0.848
No	166	164		
Yes	81	83		
Hypertension			0.144	0.704
No	165	161		
Yes	82	86		
Smoking habit			0.041	0.840
Nonsmoker	180	178		
Smoker	67	69		
Current alcohol use			0.778	0.378
No	177	168		
Yes	70	79		
Previous abdominal surgery			0.067	0.796
No	211	213		
Yes	36	34		
Chronic obstructive pulmonary disease			0.074	0.786
No	215	217		
Yes	32	30		
BMI (kg/m^2^)	23.1±3.2	22.7±2.8	1.511	0.131
Preoperative anemia			1.448	0.229
No	184	172		
Yes	63	75		
Preoperative serum albumin level (g/L)	38.4±4.0	38.2±4.3	0.680	0.497
ASA			0.495	0.482
I–II	182	175		
III–IV	65	72		
Tumor size			0.203	0.653
<5 cm	123	118		
≥5 cm	124	129		
Operation time (min)			0.980	0.322
<180	115	126		
≥180	132	121		
Blood loss (ml)	171.4±86.0	173.7±79.2	− 0.305	0.760
Grade of differentiation			1.149	0.563
Well	47	46		
Moderate	119	109		
Poorly	81	92		
Lymph node resected	24.3±11.0	24.9±9.2	− 0.652	0.515
pTNM stage			1.354	0.508
I	49	50		
II	95	106		
III	103	91		

*LC* left colectomy, *RC* right colectomy, *BMI* body mass index, *ASA* American Society of Anesthesiologists

### Comparison of the return of bowel function and length of postoperative hospital stay between the two groups after matching

The occurrence probability of PPOI in patients who had RC was 21.9% (54/247), while the occurrence probability of PPOI in patients who had LC was 13.0% (32/247). The time of first flatus, semi-liquid time, and length of postoperative hospital stay in patients who had LC were lower than those in patients who had RC (*p*<0.05) (Table 7).

**Table 7:** Comparison of postoperative gastrointestinal function recovery and discharge time between the two groups

Group	LC (*n*=247)	RC (*n*=247)	*t*/*χ*^2^	*p* value
PPOI			6.814	0.009
No	215	193		
Yes	32	54		
First flatus (days)	2.8±1.7	3.7±2.4	− 4.721	0.000
First semi-liquid diet (days)	4.3±1.7	5.3±2.4	− 5.067	0.000
Postoperative length of stay (days)	6.3±1.5	7.4±2.2	− 6.147	0.000

*LC* left colectomy, *RC* right colectomy, *PPOI* prolonged postoperative ileus

## Discussion

Compared with open colectomy, laparoscopic colectomy has many advantages, such as faster postoperative return of bowel function, fewer complications, less pain, fewer hospital stays, and similar long-term effect [16–18]. The clinical practice guideline for enhanced recovery after colon and rectal surgery from the ASCRS and SAGES strongly recommends employing a minimally invasive surgical approach whenever expertise is available and appropriate for colon cancer [8]. Laparoscopic colectomy has been widely recognized worldwide. Since 2016, most patients with curable colon cancer have undergone laparoscopic surgery in our hospital. Patients who underwent laparoscopic surgery were included in this study to eliminate errors and improve the reliability of research.

Murphy et al. [19] studied 9734 patients identified from the colectomy-specific American College of Surgeons National Surgical Quality Improvement Program database who underwent elective surgery. They found that 1364 (14%) patients had PPOI. Alhashemi et al. [14] found that the incidence of PPOI was 19% after colorectal surgery in the context of enhanced recovery pathways. The incidence of PPOI is 10.3% in a meta-analysis comprising 18,983 patients with colon cancer [20]. This is similar to the 13.1% overall incidence of PPOI in our study. However, a snapshot, prospective, observational study in England showed that the rate of PPOI was as high as 22.5% after elective colorectal surgery [21]. The reason may be that perioperative management was not performed in accordance with the ERAS guidelines in this study.

As early as 1990, Bufill confirmed the difference between right and left colon cancer from the perspective of molecular genetics and proposed that they can be regarded as distinct disease entities for the first time [22]. Subsequently, a large number of studies showed significant differences between them, such as clinical features, epidemiological features, histological characteristics, molecular biology, treatment, and prognosis [23–25]. In this study, we found statistical differences in age, sex, BMI, preoperative serum albumin level, and grade of differentiation among the two groups. Although not strongly supported by the references, the surgery of LC is more challenging to perform than that of RC due to better exposure and intraperitoneal location of the anastomosis, especially when splenic flexure mobilization or rectal mobilization is warranted [26]. In this study, the operation time of LC was longer than that of RC, indicating that LC surgery may be more difficult.

Although postoperative recovery following colorectal surgery has been well studied, few studies have directly compared the incidence of PPOI and recovery from RC and LC. The first flatus, first semi-liquid diet, and PPOI of patients who had RC are higher than those of patients who had LC. Moreover, this study showed that age, sex, smoking history, smoking habit, preoperative serum albumin level, operation type, and operation time were independent risk factors for PPOI. Therefore, propensity score matching was used to reduce the deviation caused by interference factors and make the baseline characteristics of the two groups more comparable. After matching, the differences in clinicopathologic features between the two groups were not statistically significant. The first flatus, first semi-liquid diet, length of postoperative hospital stays, and PPOI in patients who had RC were higher than those of patients who had LC. It shows that the return of bowel function after RC surgery is slower and the incidence of PPOI is higher than that in patients who had LC. Consistent with previously published studies, Garfinkle et al. [13] conducted a study based on 40,636 patients who had colon surgery in the public database of the American College of Surgeons and concluded that the PPOI of patients who had RC was 35% higher than the PPOI of those who had LC (OR=1.35, 95% CI: 1.25–1.47). The RC group also had a longer mean length of stay and more 30-day readmissions. Yuan et al. [11] studied 94 consecutive patients undergoing elective colorectal resections with primary anastomosis and found that LC results in a faster return of bowel function than RC. Grass et al. [12] found that the operative time of LC was longer than that of RC, but the incidence of PPOI in LC was lower than that in RC.

The pathophysiological mechanism underlying the slower recovery of bowel function and higher incidences of PPOI in RC has not been fully clarified. One possible explanation is that the most distal region of the colon has a specialized “rectosigmoid brake” role, with retrograde cyclic motor patterns (CMPs) occurring prominently after meals at a rate of approximately 2–4 cycles per minute, which limits rectal filling and thereby potentially contributes to continence [27]. A recent manometry study in patients who had RC showed that the distal colon becomes hyperactive after surgery with CMPs [28]. Another explanation could be that the colonic peristalsis and transit are regulated by the ileocecal valve. The ileocecal valve’s loss during RC and bowel disturbances are due to bacterial translocation from the colon to the small intestine [29, 30]. A third explanation could be the increased trauma to the small bowel associated with an ileocolic anastomosis compared to a more distal anastomosis. Surgical trauma results in a surge in sympathetic and adrenergic motor neuron activity, which causes intestinal analysis [31]. Other authors have also cited differences in vagal innervation between the proximal colon (inputs from the brainstem) and distal colon (inputs from pelvic ganglia). It has been proposed that the “pacemaker” regions in the distal colon contribute to the normal regulation of the intestinal transit, and resection of these may lead to accelerated postoperative transit through the distal colon [21].

The surgical procedure for RC is not standardized, including LRC and TLRC. TLRC was associated with significantly faster to first flatus, first semi-liquid diet, and postoperative length of stay, confirming that TLRC leads to faster recovery of bowel functions after surgery. LRC requires extracorporeal anastomosis, while TLRC requires directly intracorporeal anastomosis. TLRC with a lesser bowel mobilization, manipulation, and traction could lead to faster recovery of bowel function [32].

## Conclusion

Although ERAS has been widely accepted and popularized, PPOI remains an incurable complication after colectomy. The return of bowel function in patients who had RC was slower than those who had LC, and the incidence of RC’s PPOI was relatively higher. ERAS requires normal oral intake to be restored as soon as possible, but it may be necessary to appropriately extend the fasting time for patients who had RC.

## Data Availability

Data are available on request from the authors.

## Abbreviations

POI: Postoperative ileus
PPOI: Prolonged postoperative ileus
ERAS: Enhanced recovery after surgery
ASCRS: American Society of Colon and Rectal Surgeons
SAGES: Society of American Gastrointestinal and Endoscopic Surgeons
RC: Right colectomy
LC: Left colectomy
OR: Odds ratio
CMPs: Cyclic motor patterns
